# Postnatal zinc deficiency due to giardiasis disrupts hippocampal and cerebellar development

**DOI:** 10.1371/journal.pntd.0012302

**Published:** 2024-07-01

**Authors:** Angélica González Maciel, Laura Elizabeth Rosas López, Rosa María Romero-Velázquez, Andrea Ramos-Morales, Martha Ponce-Macotela, David Calderón-Guzmán, Francisca Trujillo-Jiménez, Alfonso Alfaro-Rodríguez, Rafael Reynoso-Robles

**Affiliations:** 1 Laboratory of Cell and Tissue Morphology, Instituto Nacional de Pediatría, Secretaría de Salud, Mexico City, Mexico; 2 Laboratory of Experimental Parasitology, Instituto Nacional de Pediatría, Secretaría de Salud, Mexico City, Mexico; 3 Laboratory of Neuroscience, Instituto Nacional de Pediatría, Secretaría de Salud, Mexico City, Mexico; 4 Laboratorio de Pharmacology, Instituto Nacional de Pediatría, Secretaría de Salud. Mexico City, Mexico; 5 Division of Neurosciences, Instituto Nacional de Rehabilitación, “Luis Guillermo Ibarra Ibarra”, Secretaría de Salud, Mexico City, Mexico; Hospital General Dr Manuel Gea Gonzalez, MEXICO

## Abstract

**Background:**

Giardiasis and zinc deficiency have been identified as serious health problems worldwide. Although Zn depletion is known to occur in giardiasis, no work has investigated whether changes occur in brain structures.

**Methods:**

Three groups of gerbils were used: control (1), orogastrically inoculated on day 3 after birth with trophozoites of two isolates of Giardia intestinalis (HGINV/WB) group (2 and 3). Estimates were made at five ages covering: establishment of infection, *Giardia* population growth, natural parasite clearance and a post-infection age. QuantiChrome zinc assay kit, cresyl violet staining and TUNEL technique were used.

**Results:**

A significant decrease (p<0.01) in tissue zinc was observed and persisted after infection. Cytoarchitectural changes were observed in 75% of gerbils in the HGINV or WB groups. Ectopic pyramidal neurons were found in the *cornus ammonis* (CA1-CA3). At 60 and 90 days of age loss of lamination was clearly visible in CA1. In the dentate gyrus (DG), thinning of the dorsal lamina and abnormal thickening of the ventral lamina were observed from 30 days of age. In the cerebellum, we found an increase (p<0.01) in the thickness of the external granular layer (EGL) at 14 days of age that persisted until day 21 (C 3 ± 0.3 μm; HGINV 37 ± 5 μm; WB 28 ± 3 μm); Purkinje cell population estimation showed a significant decrease; a large number of apoptotic somas were observed scattered in the molecular layer; in 60 and 90 days old gerbils we found granular cell heterotopia and Purkinje cell ectopia. The pattern of apoptosis was different in the cerebellum and hippocampus of parasitized gerbils.

**Conclusion:**

The morphological changes found suggest that neuronal migration is affected by zinc depletion caused by giardiasis in early postnatal life; for the first time, the link between giardiasis-zinc depletion and damaged brain structures is shown. This damage may explain the psychomotor/cognitive delay associated with giardiasis. These findings are alarming. Alterations in zinc metabolism and signalling are known to be involved in many brain disorders, including autism.

## Author summary

*Giardia intestinalis* (Syn. *G*. *duodenalis or G*. *lamblia*), is a protozoan flagellate that parasitizes humans and animals and is transmitted by the ingestion of food or water contaminated with cysts or via person-to-person contact. Causes diarrhoea. Affects children and adults worldwide. Giardiasis is known to have long-term effects. When giardiasis occurs in the first two years of a child’s life, it is associated with stunted growth and psychomotor and cognitive retardation.

Giardiasis and zinc deficiency have been identified as serious health problems worldwide. Although Zn depletion is known to occur in giardiasis, no work has investigated whether changes occur in brain structures.

We modelled *Giardia* infection in gerbils in the first days of postnatal life and found morphological changes that suggest neuronal migration is affected by zinc depletion caused by giardiasis in early postnatal life.

For the first time, the link between giardiasis-zinc depletion and damaged brain structures is shown. This damage may explain the psychomotor-cognitive delay associated with giardiasis.

Alterations in zinc metabolism and signalling are known to be involved in many brain disorders, including autism.

## Introduction

Giardiasis and zinc deficiency are serious health problems worldwide [1–7]. *Giardia intestinalis*, also known as *G*. *duodenalis* or *G*. *lamblia*, is a unicellular protozoan parasite that infects the upper small intestine of humans and other animals [8–11]; it is transmitted by ingestion of food or water contaminated with cysts or via person-to- person contact [9]. Clinical manifestations of *G*. *intestinalis* infection vary between individuals, ranging from acute to chronic infection, while some hosts may be asymptomatic. Patients with acute giardiasis present abdominal pain, explosive foul-smelling watery diarrhea, steatorrhea, vomiting, and nausea. Patients with chronic giardiasis present abdominal pain, diarrhea, weight loss, and malabsorption [8,12,13]. Giardiasis was included in the "Neglected Diseases Initiative" in 2004 [1]. In the developing countries alone it is estimated that about 300 million infections occur annually [14]. About 23.8% of children under 5 years of age infected with *Giardia* in the first 2 years of life have stunting [15,16–19], and long-term psychomotor and cognitive developmental delay [3,19,20–23]. In Mexico, an epidemiological analysis showed a seroprevalence of 55% in the general population [17]. Although *G*. *intestinalis* is common worldwide due to globalization, it is still more common in places where there is poor sanitation [18,16]. Zinc deficiency affects about 16% of the world’s population [24], with a global prevalence estimated at 31%, ranging from 4 to 73% in developing countries [25]; It is estimated that 82% of pregnant women worldwide consume less than the recommended amount of zinc [26]. In Mexico, the reported incidence of zinc deficiency is: 57% in women of reproductive age, 24% in children under 5 years and 33% in children under 2 years [27–29]. Zinc is a key trace element in biological processes in the human body. It is involved in cell differentiation, proliferation, migration and apoptosis [30] and is necessary for neuronal function [31]. Bioinformatics studies have identified nearly 3000 human proteins that are predicted to bind zinc [32]. Zinc fingers and zinc finger-containing domains require the divalent cation as a stabilizer, others require zinc for catalytic and regulatory functions of metalloenzymes [32,33]. Given the many functions that zinc plays in the immune system, a deficiency can lead to a variety of diseases [34]. Zinc is easily depleted in infectious diseases because it cannot be stored in the body [35,36]. If zinc is an essential element involved in a wide range of physiological processes, its depletion during a critical period of neurodevelopment could affect associated structures with movement and learning. Although giardiasis is known to reduce zinc [35–41], no work has investigated the status of brain structures; changes in brain structures due to giardiasis could be the origin of the long-term psychomotor and cognitive delay mentioned. The aim of this work was to confirm that giardiasis reduces zinc in addition to determining whether changes occur in the cytoarchitecture of the hippocampus and cerebellum, and to assess apoptosis.

## Methods

### Ethics statement

The animals were treated according to the Servicio Nacional de Sanidad, Inocuidad y Calidad Agroalimentaria, norma oficial 2001.NOM-062-ZOO; 1999 ([42], available at: http://www.gob.mx/senasica/documentos/nom-062-zoo-1999/. Accessed 30 Aug 2023); the internal guidelines of the vivarium of the National Institute of Pediatrics and the National Research Council (US) Committee for the Update of the Guide for the Care and Use of Laboratory Animals [43]. The Guide for the Care and Use of Laboratory Animals. 8th ed. (Washington (DC): National Academies Press (US); 2011. PMID: 21595115). The experimental procedures are part of projects approved by the Institutional Research Committees of the National Institute of Paediatrics (INP: 061/2008; 056/2012; 041/2016).

### *Giardia* culture

Trophozoites of isolate INP220806-HGINV (Human *Giardia* Invader, HGINV), belonging to assemblage A, genetic group A [44,45] and the strain reference WB (genetic group A-1) were axenically cultured in TYI-S-33 medium, harvested in log phase, washed in phosphate buffered saline (PBS, 0.1M, pH 7.0) and counted in a Neubauer chamber to obtain aliquots for inoculation gerbils [46]. Two isolates were used to see if they produced similar changes. The WB isolate is a reference strain and INP220806-HGINV was isolated from a paediatric patient. [44,45]. The concern arose because we have shown in previous work that the isolate INP220806-HGINV is able to invade the intestinal epithelium. [44,46].

### Animals

*Meriones unguiculatus* were obtained from the Biotherium of the Instituto Nacional de Nutrición “Salvador Subirán”. Gerbils were housed in Plexiglas boxes under standard vivarium conditions: a 12:12 light/dark cycle, 40% humidity and controlled temperature (23±3°C). Filtered water and commercial rodent feed were provided *ad libitum*. All feed was autoclaved and the diet was supplemented with previously disinfected sunflower seeds and carrots. To obtain pups, 9 pairs of unrelated 45-day-old pathogen-free gerbils were formed and the pairs were divided into 3 groups of 3 pairs each. Vaginal smears were taken daily from day 70 to determine the onset of gestation; the presence of spermatozoa indicated the first day of gestation.

### Gerbils infected with *Giardia* trophozoites

The experimental groups consisted of three or four animals from each litter. From previous studies we know that 90% of gerbils are infected with this protocol [46,47]. The control group (CG) contained 8 animals, and the experimental animal groups (HGINV and WB) of each age contained 11 animals per group. The pups were orally infected 3 days after birth by gavage with 1 × 10^6^ trophozoites of the HGINV or WB isolates in 50 μl of PBS (0.1 M, pH 7.4), which was used as vehicle. Control pups received 50 μl of PBS. The duodenum and brain of gerbils were examined at 11, 18, 27, 57, and 87 days post-inoculation (p. i). These times corresponded to 14, 21, 30, 60 and 90 days of age; from previous studies we know that these ages encompass the period of infection establishment, population growth and natural parasite elimination (14, 21, 30 and 60 days) and a post-infection age [46,47]. Animals were weaned on day 21, and the remaining offspring from the different litters of the same condition were housed in boxes until they reached 30, 60 and 90 days of age. Body weights of each gerbil were recorded before sacrifice and brain weights after euthanized; gerbils were deeply anesthetized with sodium pentobarbital (55 mg/kg body weight). Tissues from 8 gerbils of each group and age were used for the different procedures. The presence of trophozoites was confirmed in the duodenum of all 8 gerbils from the HGINV or WB groups. We examined changes in the hippocampus and cerebellum for two reasons: 1) they are structures associated with memory-learning and movement, respectively; 2) it is easy to detect changes in cytoarchitecture due to their laminar structure.

### Zinc in tissues

Hippocampus, cerebellum and 2 cm of duodenum were collected and frozen for subsequent zinc determination. Frozen tissues were homogenized in 200 μl of chelated hypotonic buffer, tissue samples were sonicated, centrifuged at 10,000 x g (4°C) and the supernatant was analyzed using the QuantiChrome Zinc Assay kit (BioAssay Systems) according to the manufacturer’s instructions [48,49]. This zinc assay is a colorimetric assay based on the binding of zinc to a chromogen reporting at 425 nm. Samples were assayed in duplicate using the 96-well protocol. The results were read on the microplate reader (Perkin Elmer Spectrometer).

### Histology and apoptosis

Gerbils were transcardially perfused with a cold solution of phosphate-buffered saline (PBS), 0.1 M pH 7.4, for 3 minutes, followed by a cold fixative solution containing 4% paraformaldehyde dissolved in PBS. The brains were removed and post-fixed in the same fixative for 24 h. The brains were then embedded in paraffin and 6 μm thick sections were cut with a microtome and collected on charged slides. Serial sections were taken from the dorsal hippocampus at 50 μm intervals with coordinates between -2.5 and 3.8 ± 0.15 mm from the bregma [50], and mid-sagittal sections from the cerebellum. Sections were taken 50 μm and 120 μm apart between each section to ensure that observations and estimates of hippocampal and cerebellar cells were made on sections containing different cells. For histological examination, sections were stained with cresyl violet (0.1%) [51], dehydrated with alcohol, cleared with xylol and mounted with Entellan resin (Merck, Darmstadt, Germany). To assess cell death, sections were processed using the terminal deoxynucleotidyl transferase dUTP nick end labeling (TUNEL) assay to label apoptotic cells (In Situ Cell Death Detection Kit, Roche, Mannheim, Germany) according to the manufacturer’s protocol [52–54]. Deparaffinized sections were rehydrated and then incubated with proteinase K (100 μg/mL), rinsed, incubated in H_2_O_2_ (3%), permeabilized with Triton X-100 (0.5%), rinsed again, and incubated in TUNEL reaction mixture. Sections were then rinsed and visualized using a converter-POD (horseradish peroxidase) with 3, 3′-diaminobenzidine (0.03%) (DAB). Methyl green (Sigma-Aldrich) was used as counterstain and the sections were mounted with Entellan resin.

### Quantitative analysis

Tissues were observed using an Axioskop 2 Plus microscope coupled to an image analysis system. The number of total (Cresyl violet staining) and apoptotic positive cells in a constant area (10,000 μm^2^) was estimated at a minimum of 128 frames, 40x using Zen 2.3 software. In 4 fields per section (4) for each animal (8) age and group (4 × 4 × 8); neurons were marked by clicking on their micrographs on the monitor and automatically counted; neurons with one-third of their soma within the frame were counted [52]. Micrographs were taken of the cerebellum, *cornus ammonis* 1 and 3 (CA1, CA3) and *dentate gyrus* (DG). A well-trained observer, blinded to the samples, performed all evaluations and assigned a numeric code to each slide. For quantification of cells and labeled cells (TUNEL), the cell body was defined as the counting unit (Purkinje cells in cerebellum, pyramidal cells in CA1 and CA3 and granule cells in DG). A well-trained observer, blinded to the samples, performed all evaluations and assigned a numeric code to each slide.

### Statistical analysis

Statistical analysis was performed with the inerstl 13 statistical program. First, the mean and standard deviation (SD) were calculated; normality was determined using the Shapiro Wilks test, and homogeneity using Levene’s test. The parametric procedure (ANOVA) was applied and the means were compared using Tukey’s test; considering the differences between the variances of the number of cells in the different areas in the control and experimental subjects. Statistical significance was set at p < 0.05. Data are expressed as mean values ± SD.

## Results

### Brain and body weight of gerbils

Diarrhoea was not observed in any of the cases throughout the study, but body weights of gerbils inoculated with the WB reference strain and HGINV isolates were lower than those of the control group at 14, 21 and 30 days of age, (p<0.01). Weight loss was similar between gerbils infected with WB or HGINV (Table 1). Brain weight of infected gerbils was significantly lower with both isolates at 14 days of age with both isolates; in gerbils infected with the WB isolate, a decrease was also observed at 60 days of age (Table 1).

**Table 1:** Body and brain weights of control and experimental gerbils.

Age in days	Body weight (grams)			Brain weight (grams)		
	CONTROL	WB	HGINV	CONTROL	WB	HGINV
14	15 ± 0.7	10 ± 1.0 *	11 ± 1.0 *	0.67 ± 0.08	0.63 ± 0.05*	0.61 ± 0.07*
21	20 ± 0.5	15 ± 2.7 *	15 ± 1.1 *	0.81 ± 0.04	0.78 ± 0.06	0.77 ± 0.09
30	32 ± 3.3	21 ± 1.5 *	23 ± 3.4 *	0.86 ± 0.07	0.86 ± 0.09	0.83 ± 0.09
60	54 ± 2.0	52 ± 3.0	51 ± 2.1	0.96 ± 0.06	0.92 ± 0.06*	0.95 ± 0.06
90	67 ± 3.5	64 ± 4.1	71 ± 5.4	0.98 ± 0.06	0.98 ± 0.06	1.0 ± 0.07

Mean and SD of body and brain weights of control gerbils and gerbils inoculated with trophozoites of two isolates: HGINV and WB. Body weights of inoculated gerbils were significantly lower in animals at 14, 21 and 30 days of age (*p<0.01). Brain weight of inoculated gerbils was significantly lower (*p < 0.01) at 14 days of age with both WB and HGINV isolates; at 60 days of age it was lower in gerbils inoculated with WB.

### Zinc

**Fig 1:**
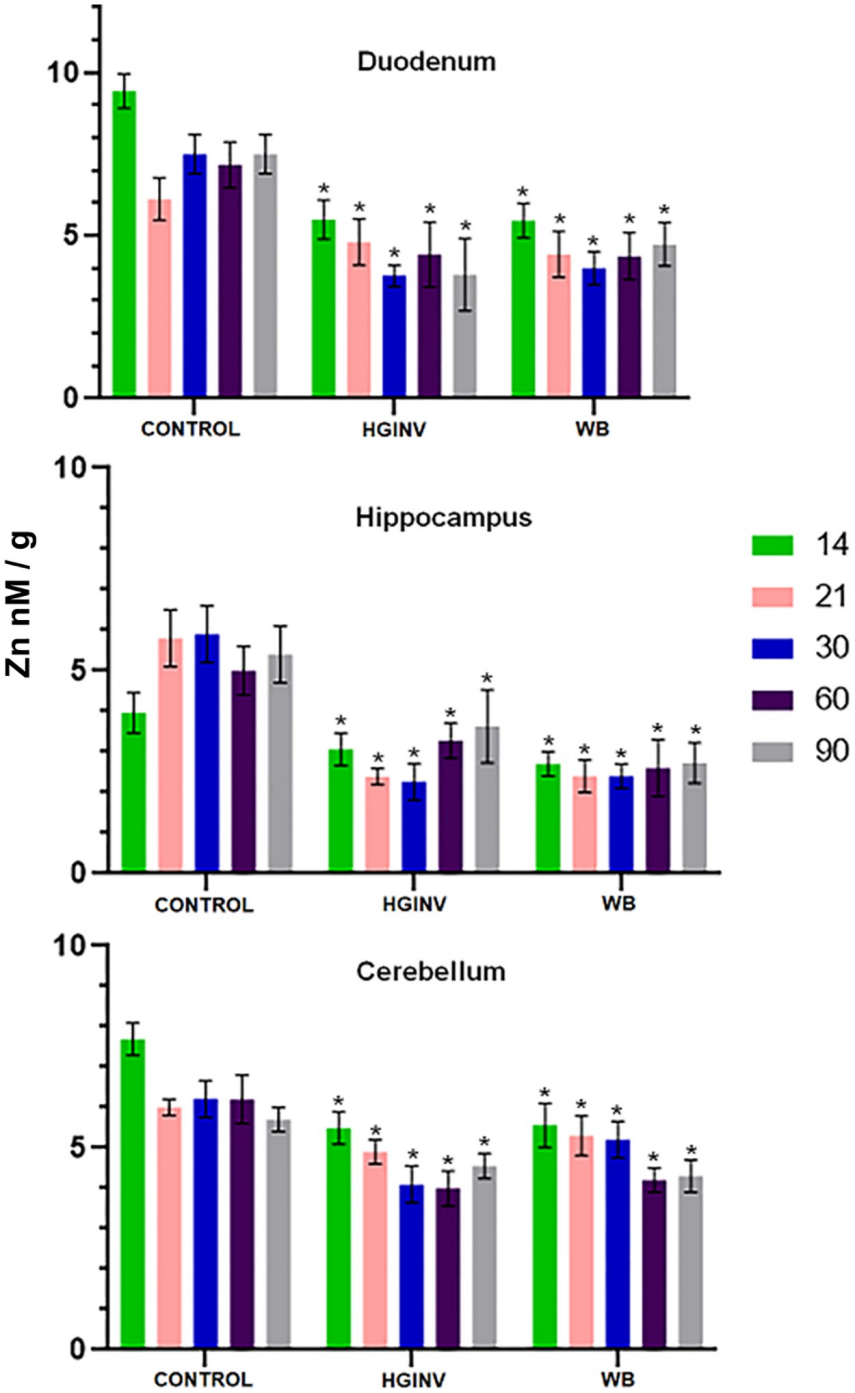
Mean and SD of zinc in tissues from control and inoculated gerbils with two isolates of *Giardia intestinalis*: WB and HGINV. A significant decrease was present in zinc levels (p<0.01) in the duodenum, hippocampus and cerebellum during the infection period from 14 to 60 days of age. The reduction was dramatic in the hippocampus. Zinc deficiency was also significant (p<0.01) in the post-infection period.

In the duodenum of the control group, Zn was highest on day 14, decreased on day 21 and was similar at 30, 60 and 90 days of age (Fig 1). In the hippocampus, Zn was lowest on day 14, increased on day 21, and remained unchanged until 90 days of age. In the cerebellum of the control group, Zn was highest on day 14, decreased on day 21, and remained similar until 90 days of age. In all three gerbil tissues of both experimental groups, we found a significant (p<0.01) decrease in zinc at all ages and even after infection (Fig 1).

### Morphology Cerebellum

**Fig 2:**
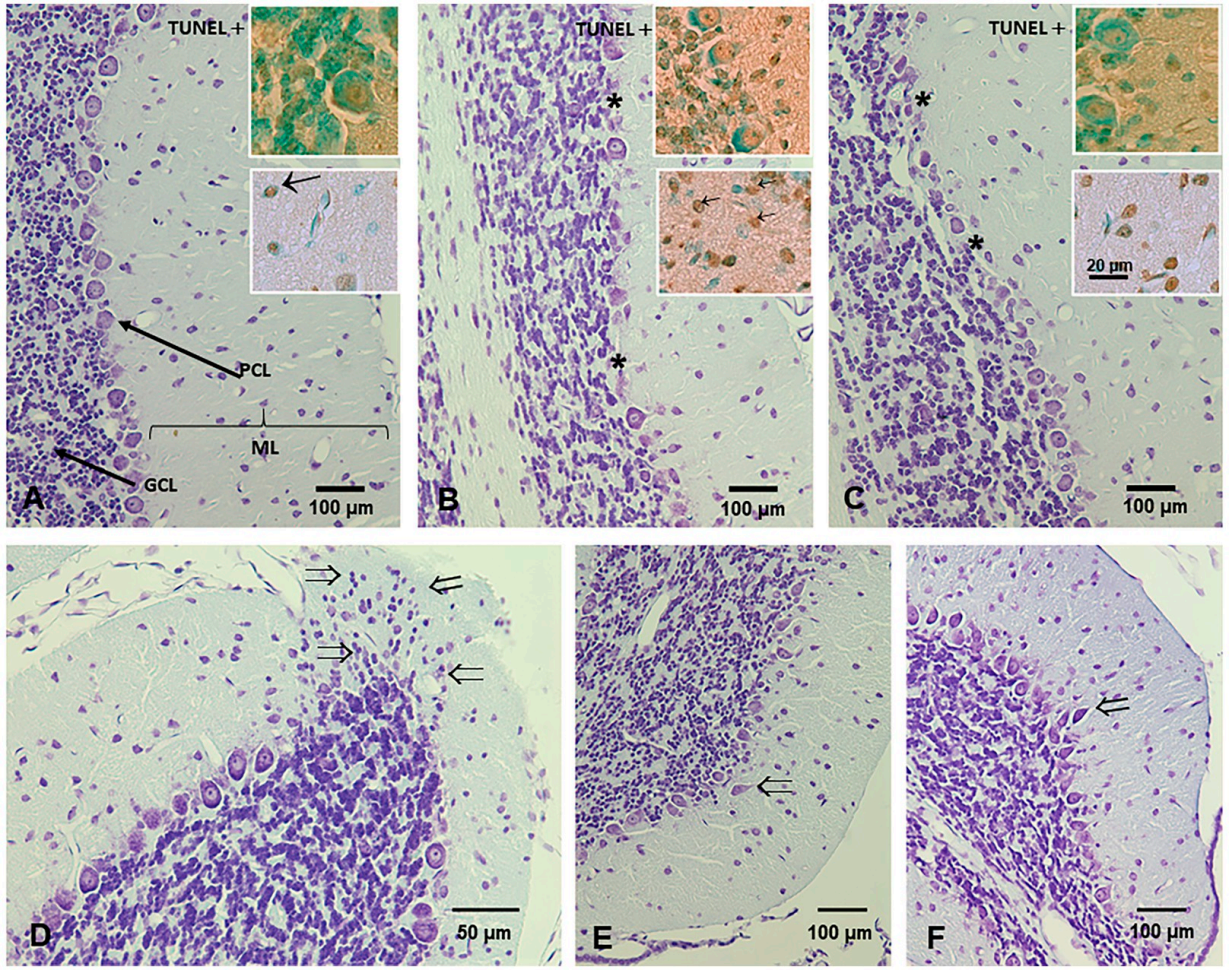
Photomicrographs of cerebellum from control and *Giardia* parasitized gerbils. **A**) Normal cytoarchitecture of the control gerbil cerebellum: molecular layer (ML), Purkinje cell (PCL) and granule cell layer (GCL). Note the large areas without Purkinje cells in B and C (*). **B**) Group HGINV gerbil cerebellum; **C**) infected with the WB isolate. The top inset shows apoptosis of Purkinje cells and the bottom inset shows apoptosis of cells present in the molecular layer. The increase in the number of TUNEL-positive cells in B and C is evident. **D**) Representative micrographs of granule cell heterotopia (⇒); **E, F**) Representative micrographs of Purkinje cell ectopia (⇒) found in the cerebellum of parasitized gerbils. A-D: 60 days of age; E-F: 90 days of age. Cresyl Violet stain; Inset: TUNEL assay, counterstain with methyl green. Magnification A, B, C, E, F = 10x; D = 20x. Inset = 40X.

The cerebellar cortex has a regular structure consisting of three layers. The outermost, beneath the pial membrane, is the molecular layer [55]; the Purkinje cell layer or ganglion layer contains the Purkinje cell somata (PCs); the deepest layer, the granule cell layer, contains the cell bodies of the granule cells [56]. On day 14, we find an outer granular layer with a thickness of four to six cells (23 ± 2 μm), the molecular layer with scattered neuronal somas, followed by the Purkinje neuron layer and the granular cell layer with a large number of tightly packed hyperchromatic nuclei. At day 21 and up to 90 days of age, the adult cerebellum appears with three layers: molecular, Purkinje neurons and granular neurons (Fig 2A, control group).

We found large areas of necrotic neurons or absence of Purkinje neurons more evident at 60 and 90 days of age in the HGINV and WB gerbils (Fig 2B and 2C), a higher density of scattered somas in the molecular layer at all ages many of them apoptotic (inset in Fig 2A, 2B and 2C). In the 60 and 90 day-old HGINV group we found granule cell heterotopia (Fig 2D) and Purkinje cell ectopy (Fig 2E and 2F).

**Fig 3:**
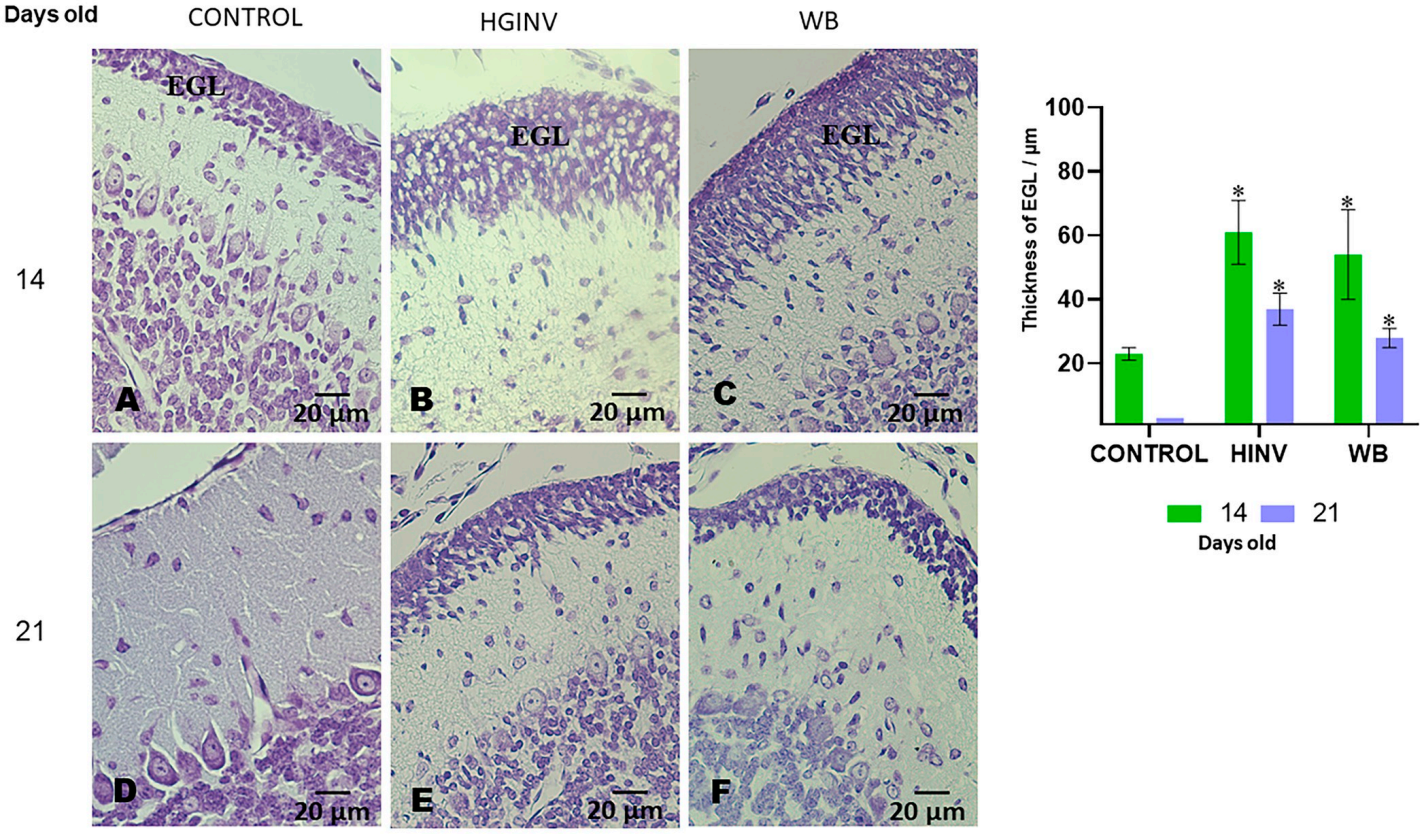
Representative photomicrographs of the cerebellum of control and *Giardia intestinalis* parasitized gerbils; Cresyl Violet stain. Thickening (mean and SD) of the external granule layer (**EGL**) is evident in 14 and 21 day-old parasitized gerbils. (Photo and graph). Magnification = 40x. Bar scale = 20 μm.

In the groups infected with *Giardia intestinalis* isolates, a significant increase (p<0.01) in the thickness (μm) of the external granular layer (EGL) was observed; at 14 days of age 61 ± 10 μm in HGINV and 54 ± 14 μm in WB; at 21 days of age, EGL remained at 37 ± 5 μm and 28 ± 3 μm, respectively (Fig 3).

**Fig 4:**
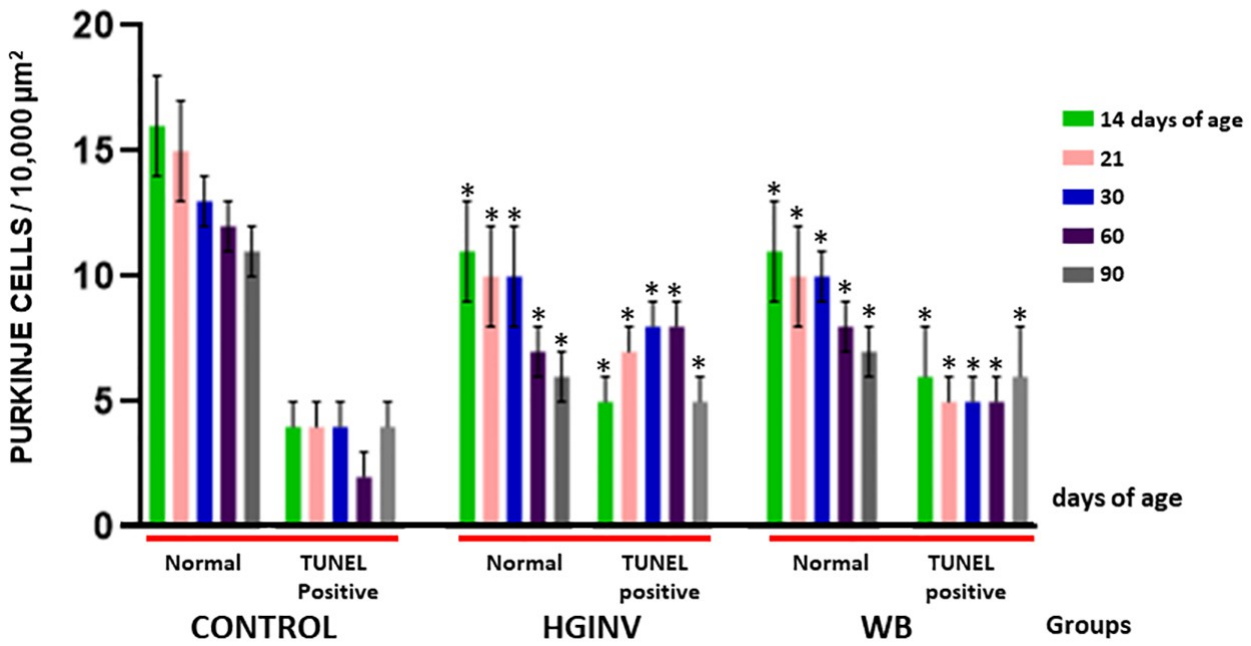
Estimation of Purkinje neurons in mid-sagittal slices of the cerebellum of control and *Giardia intestinalis* parasitized gerbils by Cresyl violet staining (Normal) and the TUNEL technique (mean and SD).

Estimation of the Purkinje cell population at all ages showed a significant decrease and the TUNEL technique showed an increase of apoptotic Purkinje neurons in the infected groups during and after infection (Fig 4).

### Hippocampus

In CA1 and CA3 regions of the hippocampus of control gerbils we found normal laminar cytoarchitecture at all ages. We found more apoptotic neurons at 14 days of age, similar at days 21 and 30 days, and decreased apoptosis at 60 and 90 days of age (Table 2).

**Table 2:** Apoptosis in CA1 and CA3.

Age (days)	CONTROL CA1-3	WB CA1-3	HGINV CA1-3
**14**	**101 ± 21**	**122 ± 38** *	**120 ± 32** *
**21**	**78 ± 35**	**81 ± 18**	**86 ± 22**
**30**	**80 ± 24**	**78 ± 19**	**105 ± 25** *
**60**	**53 ± 14**	**61 ± 16** *	**59 ± 20** *
**90**	**54 ± 12**	**66 ± 21** *	**64 ± 15** *

Mean and SD of TUNEL-positive pyramidal neurons in CA1 and CA3 regions of the hippocampus.

* = Significant increase, p < 0.01. Control; Strain WB; strain HGINV.

**Fig 5:**
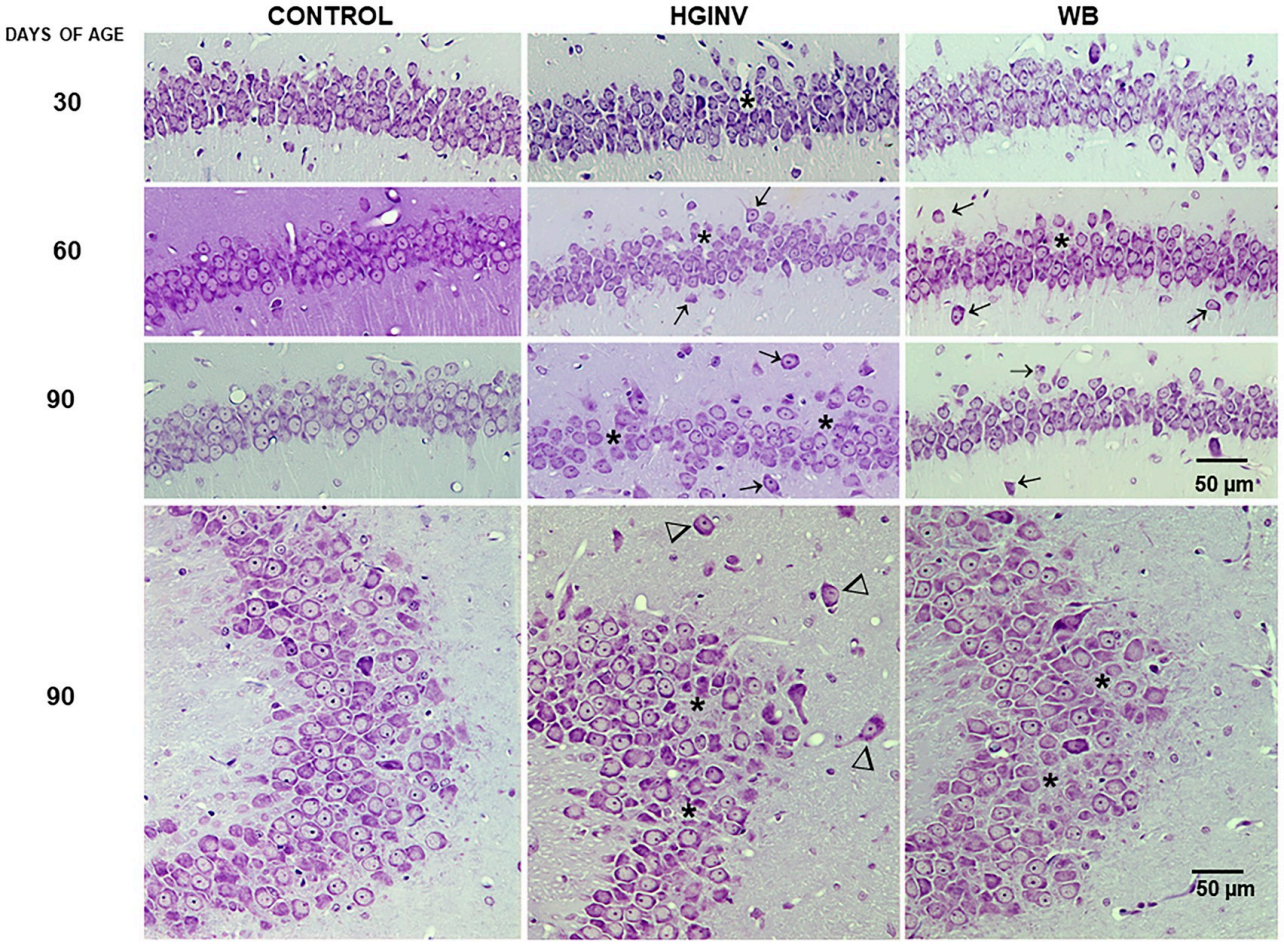
Representative micrographs of CA1 and CA3 from control, parasitized and post-*Giardia*-infected gerbils (HGINV and WB). In both CA1 and CA3 we observed an altered lamination pattern due to the absence (*), dispersal (↓) and ectopia (∇) of pyramidal neurons (HGINV). Cresyl Violet stain. Magnification = 20x. Bar scale = 50 μm.

**Fig 6:**
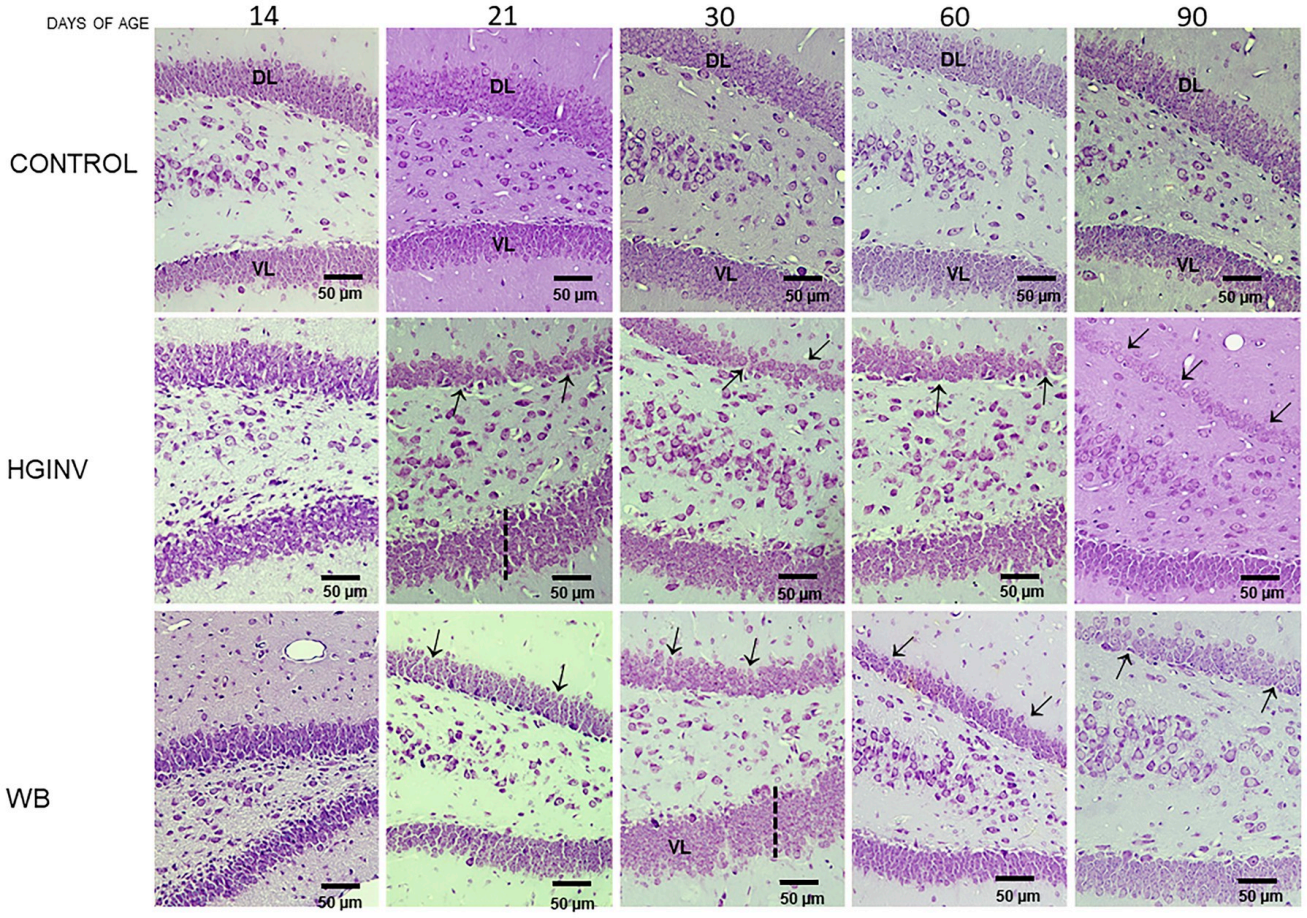
Photomicrographs of DG from control gerbils and during and after infection with *Giardia*. The typical dorsal and ventral lamination of the DG was altered in parasitized gerbils. In parasitized gerbils, the granular cell columns of the dorsal lamina (**DL**) were drastically reduced (arrows) and the thickness of the ventral lamina (**VL**) was abnormally thickened (dotted line). Cresyl Violet stain. Magnification = 20x. Bar scale = 50 μm.

In 40% of the HGINV and WB gerbil groups, we found reduced, ectopic and scattered pyramidal neurons in *cornus ammonis* (CA1 and 3) at 30, 60 and 90 days of age; loss of lamination was evident in the HGINV group at 60 and 90 days of age (Fig 5). In the dentate gyrus (DG) of HGINV and WB gerbils, we found a thinning of the dorsal lamina of granular cells and, from day 30 of age, a marked abnormal thickness of the ventral lamina (Fig 6). In the parasitized groups, pyramidal and pyknotic granular cells were observed at all ages. In the WB group, apoptosis in CAs was higher (p<0.01) than in the control group at 14, 60 and 90 days of age; in the HGINV group it was higher (p<0.01) at 14, 30, 60 and 90 days of age (Table 2). In DG, the infected groups showed a significant (p<0.01) increase or decrease in apoptosis (Table 3).

**Table 3:** Apoptosis in DG.

Age (days)	CONTROL DG	WB DG	HGINV DG
**14**	**104 ± 36**	**140 ± 61** *	**136 ± 32** *
**21**	**138 ± 33**	**107 ± 38** ↓	**109 ± 50** ↓
**30**	**146 ± 25**	**139 ± 48**	**158 ± 39**
**60**	**108 ± 16**	**92 ± 12** ↓	**95 ± 14** ↓
**90**	**85 ± 32**	**110 ± 30** *	**104 ± 15** *

Mean and SD of TUNEL-positive granular cells in the DG.

* = Significant increase,

^↓^ = significant decrease, p < 0.01. Control; strain WB; Strain HGINV.

## Discussion

Giardiasis in the first two years of life is associated with delayed psychomotor development and cognitive impairment (3, 15–23). The aim of this study was to confirm whether giardiasis initiated on postnatal day 3 reduces zinc in the duodenum, hippocampus and cerebellum. The cytoarchitecture of the hippocampus and cerebellum was analyzed; these structures are related to cognition and movement respectively. Estimates were made at different ages, spanning the time of infection establishment, *Giardia* population growth and natural parasite clearance. In this model, we know that *Giardia* is naturally eliminated at postnatal day 60. The changes persist after 90 days (120 and 180 days were not included). In previous work we have demonstrated the invasive potential of *Giarda* isolate HGINV [46,47]. In this work we found that both isolates cause similar damage.

Our results showed that giardiasis, initiated at a critical period of neurodevelopment, significantly reduces zinc in all three tissues and induces changes in the cytoarchitecture of the hippocampus and cerebellum. The changes persist after infection and for a long time, so it is possible that something similar may happen in children who have had giardiasis in the first two years of life. This would be an explanation for the psychomotor and cognitive delay above.

As mentioned above, there are no similar studies. To explain our findings, we can only look to experimental studies that have been conducted to understand lamination defects.

In CA1 and CA3 regions of the hippocampus of gerbils parasitized, we found ectopic pyramidal neurons, lamination was thinner or lost due to death or dispersion of pyramidal neurons. In the cerebellum, Purkinje cells were significantly reduced.

Hippocampal and cerebellar macroneurons are generated during the embryonic stage [57–59]. The Zbtb20 gene (also known as HOF, Znf288, Zfp288) encodes two protein isoforms, designated Zbtb20S and Zbtb20L, which are expressed in newborn pyramidal neurons [60]. Zbtb20 is expressed in pyramidal neurons in CA3 and CA1 areas of the mouse hippocampus during the critical period of pyramidal neuron neurogenesis [61, 62].

To determine the origin of CA1 and CA3 striatal pyramidal lamination defects in the hippocampus, they generated transgenic mice with ectopic expression of Zbtb20S and ZbtbL in ectopic immature pyramidal neurons differentiated from non-hippocampal multipotent precursors. They found that Zbtb20 delays radial migration of immature cortical neurons and orchestrates the formation of a pyramidal cell layer, suggesting that Zbtb20 acts as a molecular switch of a pathway that induces pyramidal neuron morphogenesis and suppresses transitions of newborn neurons and their fate within the brain structure. Zbtb20 was also found to be expressed in the subventricular zone (SVZ).

The SVZ is an important neurogenic niche that contains stem cells throughout life [63]; whether they are present in the DG subgranular is unknown.

The difference in the cytoarchitecture of the laminar arrangement of CA1, CA3, and Purkinje neurons indicates that the settlement of these neurons was normal; the observed change is likely the sum of normal apoptosis and the increased necrosis and apoptosis of neurons observed at all ages in response to altered connectivity with granule neurons in postnatal development [59].

The development of hippocampal and cerebellar granule cells spans both the prenatal and early postnatal periods. Cerebellar granule neuron precursors (GNPs) are generated in the rhombic lip during embryonic development; on embryonic day 13 they begin to migrate and proliferate in the external granular layer (EGL), which is located on the surface of the cerebellar cortex [64]. The proliferation peak of GNPs is from postnatal day 7 to 10; they continue to proliferate in the EGL [64–66]; they exit the cell cycle to differentiate into granule neurons. The differentiated granule neurons migrate inward [67] beyond the Purkinje cells to form the inner granular layer (IGL). Here they extend their axons into the molecular layer to form parallel fibers [63]. Mature Bergmann glia and Purkinje neurons provide the scaffold necessary for this migration process. The depletion of EGL is complete by P18 [64–66,68].

The thickness of the outer granular layer at 14 and 21 days of age in zinc-deficient animals may be explained by a delay in the proliferation, differentiation and migration of precursor neurons from the outer granular layer and a consequent delay in their migration to the inner granular layer.

The clusters of granular neurons found in the molecular region suggest changes in proteins involved in migration.

Zinc-bouns proteins involved in migration have been identified, such as the zinc finger transcription factor GLI3, a regulator of cerebellar neuronal migration and differentiation [69], the zinc finger transcription factor RU49 [70] or zinc finger Gli-Kruppel [71].

The DG develops from precursors of the dentate neuroepithelium (DNE); neurons are generated from the DNE from embryonic day (E) 13.5 and at E17.5 these precursors migrate and accumulate within the cleft to form the neural stem cell (NSC) layer. It has been suggested that NSCs are generated perinatally in the ventral region of the dentate gyrus (VDG) and subsequently migrate to the dorsal subgranular region of the dentate gyrus (DDG); [72–76]. Neurogenesis and apoptosis peak between postnatal days 7–14 [77–79].

The greater number of granule cells in the ventral lamina suggests that NSCs generated in the ventral DG may not have differentiated and migrated to the dorsal subgranular region [70]; we suggest that zinc deficiency may have affected pathways that require zinc for differentiation and migration.

The expression of the RU49 transcription factor is known to be restricted to the three granule neuron lineages of the central nervous system, the cerebellum, dentate gyrus and olfactory bulb. It has been suggested that RU49 may play a critical role in their differentiation [70,71,80], a point that will be tested in future work.

Zinc is present in the brain as a structural component of approximately 70% of proteins, contributing to the efficient functioning of more than 2000 transcription factors and more than 300 enzymes. Around 10–15% of brain zinc occurs in “free” or “chelable” form, and is present in much lower concentrations (~500 nM) in brain extracellular fluids [81,82]. Zinc depletion due to giardiasis may have altered these and other zinc-requiring pathways, but this remains to be proven.

Zinc possesses chemical properties that make it unique and very useful in a number of biological systems, so it is involved in a large number of metabolic processes [83].

It plays an active role in the catalytic site of several important enzyme systems; unlike iron and copper, it does not change its electrochemical state, so it is not useful in oxidation-reduction reactions; however, for the same reason, the organism is not at risk of oxidative damage, allowing zinc to be easily transported and utilized. In addition to its role as a catalytic ion, zinc is also a structural ion involved in some biological membranes or nucleic acids [84]. It can form cross- links as in the bases of the so-called "zinc fingers" that characterize some transcription proteins [85,86].

Zinc is required for the integrity of histones, proteins intimately associated with DNA, and is also a component of DNA polymerases, RNA and cytosolic enzymes involved in protein synthesis. Zinc is an intracellular regulator with a biological importance similar to that of calcium [84]. Given the extraordinary diversity of biological functions of this micronutrient, we can deduce that its deficiency may affect cellular function at various sites.

To our knowledge, there have been no studies such as this describing how zinc deficiency caused by giardiasis affects brain structures.

Although we believe that the observed changes are a consequence of the myriad metabolic changes that occur as a consequence of zinc deficiency caused by giardiasis at a critical time in development, we cannot rule out the possibility that other systems involved in proliferation, differentiation and migration events during the first days of postnatal life are also affected.

We do not know the status of other elements that regulate the proliferation and differentiation of granule cells in the cerebellum and hippocampus [68, 87], or of proteins that act as effectors of movements required for neuronal migration [88–91], as well as regulators of neuronal positioning such as Reelin, which may act as a braker of migration signals [88, 92–100]. Giardiasis in the first two years of life is associated with delayed psychomotor development [19] and impaired cognition [3,19–23]. At this point in the discussion, we cannot fail to mention that zinc deficiency has been associated with many brain disorders, including autism (ASD), which is characterized by altered zinc metabolism and signaling [81, 101–106]. The core etiology of ASD is thought to be related to overlapping genetic, epigenetic, and environmental factors [101,107–110]. Epidemiological studies have shown a clear association between zinc deficiency in early life and autism spectrum disorder (ASD). In line with this, studies in mice have shown that inadequate zinc intake during pregnancy is a significant risk factor for neurobiological and behavioral abnormalities in the offspring, similar to those observed in individuals with ASD [111]; in the same context, zinc supplementation during pregnancy has been found to reduce ASD-like behavioral symptoms [112], randomized, double-blind, placebo-controlled clinical trials are considered necessary to validate the beneficial therapeutic effects of Zn in pregnant women or in very early stages of ASD and Phelan McDermid syndrome (PMDS) patients [112].

In this study, we found that zinc deficiency caused by giardiasis in early life leads to errors in neuronal migration, which is a hallmark of autistic brains [113,114]. Our findings suggest that giardiasis may be another factor contributing to the development of autism spectrum disorder (ASD). Although early life infections with Giardia have been associated with impaired cognitive function, this is the first study showing changes in the cytoarchitecture of the hippocampus and cerebellum that may underlie this deficiency. Giardiasis is a neglected disease.

## Conclusion

The results of the present study provide important information on CNS neurodevelopment in the early postnatal period. In addition, the morphological changes found suggest that neuronal migration is impaired due to zinc depletion caused by giardiasis, which may lead to alterations in zinc metabolism and signalling implicated in many brain disorders, including autism.

## Supporting information

S1 Data Contains the data from which tables and figures have been constructed: Tab A. Data for Table 1. Mean and SD of body and brain weights of control gerbils and gerbils inoculated with trophozoites of two isolates: HGINV and WB. Tab B. Data for Fig 1. Mean and SD of zinc in tissues from control and inoculated gerbils with two isolates of Giardia intestinalis: WB and HGINV. A significant decrease was present in zinc levels. Tab C. Data for Fig 3. Representative photomicrographs of the cerebellum of control and Giardia intestinalis parasitized gerbils; Cresyl Violet stain. Thickening (mean and SD) of the external granule layer (EGL) is evident in 14 and 21 day-old parasitized gerbils. Tab D. Data for Fig 4. Estimation of Purkinje neurons in mid-sagittal slices of the cerebellum of control and Giardia intestinalis parasitized gerbils by Cresyl violet staining (Normal) and the TUNEL technique (mean and SD). Tab E. Data for Table 2. Mean and SD of TUNEL-positive pyramidal neurons in CA1 and CA3 regions of the hippocampus and Table 3. Mean and SD of TUNEL-positive granular cells in the DG.
(XLSX)
